# Addition of immune checkpoint inhibitors to intravesical BCG for high‐risk BCG‐naïve non‐muscle invasive bladder cancer: Systematic review and meta‐analysis

**DOI:** 10.1002/bco2.70194

**Published:** 2026-03-31

**Authors:** Renil S. Titus, Carlos Riveros, Eusebio Luna Velasquez, David‐Dan Nguyen, Suran Somawardana, Amar Singh, Dharam Kaushik, Guru Sonpavde, Alexandre Zlotta, Girish Kulkarni, Srikala S. Sridhar, Christopher J. D. Wallis, Raj Satkunasivam

**Affiliations:** ^1^ Department of Urology Houston Methodist Hospital Houston Texas USA; ^2^ Division of Urology, Department of Surgery University of Toronto Toronto Ontario Canada; ^3^ Rice University Houston Texas USA; ^4^ Southwestern University Georgetown Texas USA; ^5^ Advent Health Cancer Institute Orlando Florida USA; ^6^ Division of Urology Mount Sinai Hospital Toronto Ontario Canada; ^7^ Division of Urology, Department of Surgery University Health Network Toronto Ontario Canada; ^8^ Division of Medical Oncology, Princess Margaret Cancer Centre, University Health Network University of Toronto Toronto Ontario Canada

**Keywords:** BCG naive, immune checkpoint inhibition, non‐muscle invasive bladder cancer, urothelial carcinoma

## Abstract

**Objective:**

The objective of this study is to assess the efficacy, safety and change in quality of life associated with ICI‐BCG (I + M) compared to BCG (I + M) in patients with BCG‐naïve HR‐NMIBC.

**Material and Methods:**

A systematic search was run on PubMed, Embase and CENTRAL from inception to 29 October 2025. Phase 3 randomized controlled trials comparing safety, efficacy and quality of life (QOL) for patients with BCG‐naïve HR‐NMIBC receiving ICI‐BCG (I + M) versus BCG (I + M): hazard ratios (HR) and 95% confidence interval (95% CI) for HG‐RFS (high‐grade recurrence free survival), odds ratio (OR) and 95% CI for Grade ≥3 treatment‐related adverse events (G3 + TRAEs), and mean difference (MD) and 95% CI for changes in quality of life (QOL measured using EORTC QLQ C30). A priori defined subgroups included presence of CIS, presence of papillary tumours, age <65 or ≥65 years, male/female, and BCG strain.

**Results:**

Pooled results indicated that ICI‐BCG (I + M) was not associated with a reduction in HG‐RFS (HR: 0.78; 95% CI: 0.59–1.02). However, on sensitivity analysis excluding ALBAN (for differences in trial design and patients included), ICI‐BCG (I + M) was associated with a reduction in HG‐RFS (HR: 0.68; 95% CI: 0.54–0.85) with an absolute risk reduction of 4.4–7.3% in 36‐month HG‐RFS. ICI‐BCG (I + M) was associated with an increased risk of G3 + TRAEs (OR: 4.76; 95% CI: 3.01–7.53). ICI‐BCG (I + M) was associated with a non‐clinically significant decline in QOL (MD: −3.25; 95% CI: −5.11 to −1.39). Heterogeneity between trials was minimal (*τ* ^2^ < 1). Risk of bias was low in all included studies.

**Conclusion:**

This pooled analysis provides data for patient‐specific counselling on the use of ICI‐BCG (I + M) for BCG‐naive HR‐NMIBC.

## 1 INTRODUCTION

Bladder cancer is the ninth most prevalent cancer, and approximately 75% of cases are non‐muscle invasive (NMIBC) [1]. Approximately half are considered high‐risk and treated by transurethral resection and intravesical induction and maintenance‐BCG (BCG‐I + M) [2]. Although it is associated with a high response rate, 30–40% of patients fail treatment, requiring other intravesical therapies or escalation of therapy for progression [3]. There remains a significant unmet need to decrease recurrence rates and improve the durability of response.

Immune checkpoint inhibitors (ICIs) have shown activity, with pembrolizumab (anti‐PD‐1) being approved for BCG‐unresponsive NMIBC [4]. Given the potential synergy between ICI and BCG [5], three independent Phase 3 randomized controlled trials (RCTs) examined the combination of BCG(I + M) with ICI (Sasanlimab, Durvalumab and Atezolizumab) [6, 7, 8]. However, their results were inconsistent, making it challenging to balance the potential benefit of recurrence risk reduction against the risk of life‐altering immune‐related adverse events. Moreover, these trials did not report a significant benefit among patients not receiving maintenance‐BCG. In the context of ongoing BCG shortages [9] and the uncertain implications of early ICI exposure on future treatment options for MIBC or metastatic disease, it is unknown if ICI‐based regimens produce a clinically meaningful effect and, if so, in what patient groups. To address these through a summative synthesis of these pivotal and potentially practice‐changing trials, we performed a systematic review (SR) and meta‐analysis (MA) to assess the efficacy and safety of ICI‐BCG (I + M) versus BCG (I + M) for patients with BCG‐naïve HR‐NMIBC in RCTs. Further, we conducted a network meta‐analysis (NMA) to compare the relative safety and efficacy of the regimens.

## 2 METHODS

These systematic review, meta‐analysis and network meta‐analysis were performed according to the Preferred Reporting Items for Systematic Reviews and Meta‐analyses (PRISMA) guidelines and PRISMA for network meta‐analysis guidelines [10, 11]. The protocol has been registered in the International Prospective Register of Systematic Reviews database (PROSPERO CRD420251178140).

### 2.1 Search strategy and study selection

Using the terms ‘randomized’, ‘randomised’, ‘immune’, ‘checkpoint’, ‘BCG’, ‘NMIBC’ and ‘non‐muscle‐invasive bladder cancer’, we searched PubMed, Embase and CENTRAL from inception to 29 October 2025. Only English‐language Phase III randomized controlled trials comparing the safety and efficacy of ICI + BCG (I + M) regimens to BCG (I + M) for BCG‐naive high risk NMIBC were included. MS Excel functions were used to remove duplicates, and studies were screened manually, independently and in duplicate. Non‐randomized trials and retrospective studies were excluded.

### 2.2 Outcomes and quality assessment

The primary outcome was high‐grade recurrence free survival (HG‐RFS). Secondary outcomes included Grade ≥3 treatment‐related adverse events (G3 + TRAEs) and decline in quality of life (QOL: measured by EORTC QLQ C30 [12]). We also assessed Grade ≥3 immune‐related adverse events (G3 + irAE) because these are of special interest and may require lifelong treatment.

Risk of bias in the included studies was assessed using the Cochrane risk‐of‐bias (RoB2) tool for randomized trials [13]. This qualitative assessment evaluates six domains: randomization, allocation, concealment, blinding of participants and personnel, blinding of outcome assessment, attrition bias and selective reporting. Each domain could be judged as having ‘low’, ‘some concerns’ or ‘high’ risk of bias.

### 2.3 Data extraction and statistical analysis

Data were extracted independently and in duplicate by two authors (RST and SS). For HG‐RFS, hazard ratios (HR) and 95% confidence intervals (95% CI) were extracted from the included studies. Absolute risk reduction (ARR) and numbers needed to treat (NNT = 1/ARR) at 36 months for HG‐RFS were derived. For G3 + TRAEs, log‐ORs with SE were computed from 2 × 2 contingency tables. For Grade ≥3 immune‐related adverse events (G3 + irAEs), attributable risk (AR) and numbers needed to harm (NNH = 1/AR) were derived. For QOL decline, mean change from baseline at end of treatment regimen was extracted, and mean differences (MDs) were pooled.

Pairwise random‐effects meta‐analyses (RE‐MA) were conducted to compare ICI‐BCG (I + M) to BCG (I + M) arm due to possible heterogeneity from ICI used, BCG regimen, geographic differences in recruitment and different proportions of patients with T1 disease and CIS. Due to the small number of trials, Restricted Maximum Likelihood (REML) was used to estimate between‐study variance (*τ* ^2^), and heterogeneity was summarized using *τ* ^2^ and *I* ^2^ statistics. Hartung–Knapp (H‐K) adjustments were used to account for the small number (<5) of trials.

A frequentist NMA was performed to compare individual ICI‐BCG (I + M) regimens using BCG (I + M) as the common comparator. Effect estimates were modelled on the log‐HR (efficacy) and log‐OR (safety) scales. RE‐NMA models using REML were applied. *p* scores (probabilistic treatment rankings) were calculated, where higher *p* scores indicated greater efficacy, lower toxicity or greater QOL‐decline. Tables and forest plots were used for visualization. MA was performed using the metagen() function (meta package) and NMA using the netmeta() package in R (RStudio environment). *p* values were two‐sided, and values <0.05 were deemed significant.

### 2.4 Subgroup and sensitivity analysis

We performed a priori‐defined subgroup analysis (1–5): (1) CIS at randomization present/absent; (2) BCG strain: TICE/other; (3) age <65 or ≥65 years; (4) sex: male/female; and (5) papillary disease at randomization present/absent.

For the sensitivity analysis, we excluded the ALBAN trial due to its smaller sample size, difference in the definition of primary endpoint, lower proportion of patients with T1 disease and CIS‐only disease included despite similar target patient population and non‐allowance of BCG reinduction for persistence of CIS at 3 months.

## 3 RESULTS

### 3.1 Characteristics and risk of bias of the included studies

**FIGURE 1:**
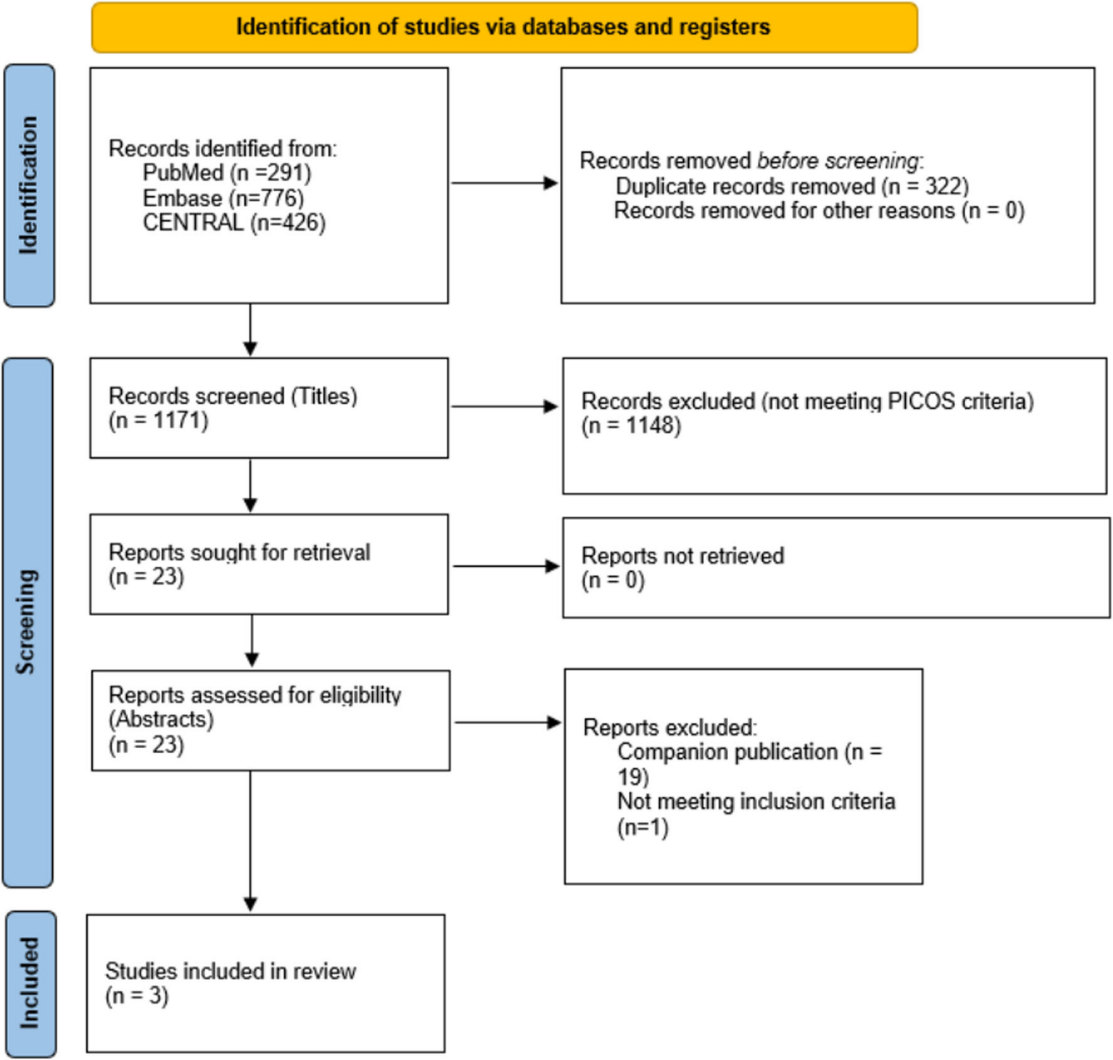
PRISMA flowchart of included studies. PICOS: patient, intervention, control, outcome, study type.

We queried 1493 records from the initial search. After two screening stages, three unique phase III RCTs with a total of 1899 patients were included (Figure 1). A summary of study characteristics is included in Table 1. The experimental arm in CREST (NCT04165317), POTOMAC (NCT03528694) and ALBAN (NCT03799835) included administration of up to 2 years of Sasanlimab, 1 year of Durvalumab and 1 year of Atezolizumab. The primary endpoints in CREST and POTOMAC were similarly defined as event‐free survival and disease‐free survival, respectively. Events in these trials included high‐grade NMIBC recurrence, progression and persistence of CIS at 6 months or death. The primary endpoint in ALBAN was event‐free survival defined to include any‐grade recurrence in NMIBC, progression, 6‐month persistence of CIS, upper tract recurrence or death. CREST and POTOMAC met their primary endpoint, whereas ALBAN did not.

**TABLE 1:** Characteristics of included studies.

Study (year)	Arms	Regimen	Participants (*N*)	Median age (years)	Female sex (%)	Number with CIS (%)	Current smoker status	Median follow‐up (months)	Primary endpoint	Secondary endpoints
ALBAN (2025)	Atezolziumab + BCG (I + M)	Atezolizumab 1200 mg q3w up to 1 year; BCG induction with 1‐year maintenance	262	67.7	41 (15.6)	106 (40.5)	50 (19.1)	35.3	EFS^a^	HG‐RFS, OS, CRR, DOR among patients with CIS, QOL, safety
	BCG (I + M)	BCG induction with 1‐year maintenance	255	67.0	34 (13.3)	96 (37.6)	38 (15.0)			
CREST (2025)	Sasanlimab + BCG (I + M)	Sasanlimab 300 mg q4w up to 25 cycles; BCG induction with 2‐year maintenance	352	67	72 (20.5)	88 (25.0)	71 (20.2)	36.3	EFS (Arm A vs.)^b^	EFS (Arm B v. C), OS, CRR among patients with CIS, QOL, safety
	Sasanlimab + BCG (I)	Sasanlimab 300 mg q4w up to 25 cycles; BCG induction only	352	67	53 (15.1)	93 (26.4)	70 (19.9)			
	BCG (I + M)	BCG induction with maintenance	351	67	67 (19.1)	88 (25.1)	54 (15.4)			
POTOMAC (2025)	Durvalumab + BCG (I + M)	Durvalumab q4w for 13 cycles; BCG induction with 1‐year maintenance	339	68	63 (18.6)	125 (36.9)	62 (18.6)	60.7	DFS (Arm A v. C)^c^	DFS (Arm B v. C), 6 month CRR among CIS patients, QOL, time to progression, time to cystectomy, time to new UTUC, any DFS, 5‐year OS, safety
	Durvalumab + BCG (I)	Durvalumab q4w for 13 cycles; BCG induction only	339	68	67 (19.8)	125 (36.9)	60 (17.7)			
	BCG (I + M)	BCG induction with maintenance	340	67	69 (20.3)	125 (36.9)	63 (18.5)			

Abbreviations: BCG (I + M), BCG induction and maintenance; CIS, carcinoma in situ; CRR, complete response rate; DFS, disease free survival; DOR, duration of response; EFS, event free survival; HG‐RFS, high‐grade recurrence free survival; OS, overall survival; QOL, quality of life; UTUC, upper tract urothelial carcinoma.

^a^ Event free survival was defined as time from randomization to the time of the first EFS event (high‐grade or low‐grade NMIBC relapse, persistence of CIS after 6 months after the start of therapy [for patients with CIS at randomization], progression of disease, appearance of upper tract urothelial carcinoma [UTUC] or death whatever the cause).

^b^ Event free survival for Arm A versus Arm C, defined as time from randomization to recurrence of high‐grade disease, progression of disease, persistence of CIS (for patients with CIS at randomization) or death due to any cause.

^c^ Disease‐free survival was defined as the time from randomization until first recurrence of high‐risk disease (recurrence of high‐risk NMIBC [high‐grade Ta, T1 or carcinoma in situ]; presentation of muscle‐invasive bladder or urothelial cancer, or metastatic disease, or both; or persistent carcinoma in situ at 6 months) or death (by any cause in the absence of recurrence).

Key differences between these trials were the inclusion of low‐grade recurrences and upper‐tract recurrences in the primary endpoint for ALBAN, whereas the others only included high‐grade recurrences. The ALBAN trial was a smaller study, with approximately 200 fewer patients across the ICI‐BCG (I + M) and BCG (I + M) arms. Although the eligibility criteria were similar across the three trials, among the included patients, the proportion of those with CIS‐only disease and high‐grade T1 disease varied between the trials. CREST (58%) and POTOMAC (60%) had a higher proportion of T1 disease compared to ALBAN (39%). ALBAN had a lower proportion of patients with only‐CIS histology (no papillary tumour) at 7% compared to CREST (15%). Regarding the treatment protocol, both CREST and POTOMAC pursued up to 2‐year maintenance‐BCG, whereas ALBAN pursued up to 1‐year maintenance‐BCG. Further, CREST and POTOMAC patients were allowed to receive BCG reinduction for persistence of CIS at 3 months. Sasanlimab was administered subcutaneously, whereas Durvalumab and Atezolizumab were delivered intravenously. The overall risk of bias for the included RCTs was low (Figure S1).

### 3.2 Pooled analysis of HG‐RFS

**FIGURE 2:**
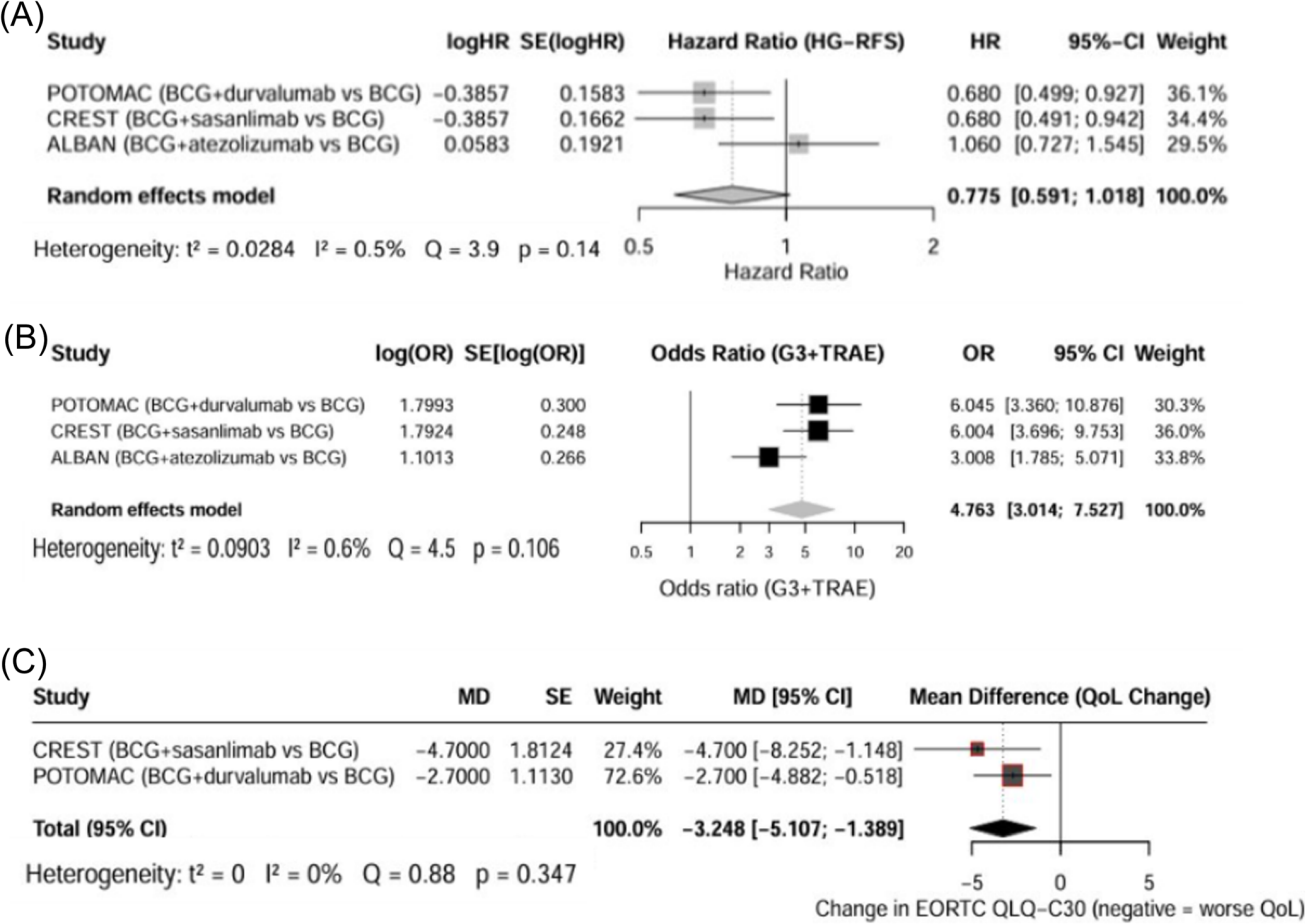
Forest plot for meta‐analysis of HG‐RFS (A), Grade ≥3 TRAE (B) and QOL (C). EORTC QLQ C30, European Organisation for Research and Treatment of Cancer Quality of Life Questionnaire Core 30; HG‐RFS, high grade recurrence free survival; HR, hazard ratio; MD, mean differences; QOL, quality of life; TRAE, treatment related events; 95% CI, 95% confidence interval.

Pooling results for HG‐RFS did not find significant heterogeneity between the studies (*τ* ^2^ = 0.017). In this analysis, ICIs were not associated with a statistically significant benefit in HG‐RFS overall (HR: 0.78; 95% CI: 0.59–1.02; Figure 2). The improvement was statistically significant in the sensitivity analysis excluding ALBAN (HR: 0.68; 95% CI: 0.54–0.85; Figure S2). The ARR at 36 months for HG‐RFS in CREST was 7.3% (ICI‐BCG vs. BCG: 82.1% vs. 74.8%; NNT ≈ 14) and in POTOMAC was 4.4% (ICI‐BCG vs. BCG: 81.8% vs. 77.4%; NNT ≈ 23). NMA evaluating whether any specific regimen offered a more favourable balance of benefit‐to‐harm indicated that Durvalumab + BCG (I + M) and Sasanlimab + BCG (I + M) had comparable efficacy and similar odds of G3 + TRAEs (Figure S3 and Table S1).

### 3.3 Subgroup analysis

ALBAN was excluded from subgroup analysis because data regarding HG‐RFS for subgroups were not available. In subgroup analyses, there was a significant benefit irrespective of age though the association of improved HG‐RFS was restricted to patients without CIS disease at randomization (HR 0.64; 95% CI: 0.45–0.91), using non‐TICE strain (HR 0.63; 95% CI: 0.47–0.83) and male patients (HR 0.65; 95% CI: 0.51–0.83). The benefit associated with ICI‐BCG (I + M) versus BCG (I + M) among other subgroups tended towards improvement in HG‐RFS but was not statistically significant. These may reflect the conservative estimation by H‐K adjustment or reduced power from small sample sizes (Figure S4).

### 3.4 Pooled analysis of secondary outcomes

On pooled analysis, ICI‐BCG (I + M) was associated with greater odds of G3 + TRAEs (OR: 4.77; 95% CI: 3.01–7.53, *τ* ^2^ = 0.0903; Figure 1) and decline in EORTC QLQ C30 score (MD –3.25; 95% CI: −5.11 to −1.39; *τ* ^2^ = 0; Figure 1). For G3 + irAE, the AR was 15.7% in CREST (NNH ≈ 6) and 7.7% in POTOMAC (NNH ≈ 13), indicating that for every 6–13 patients treated with ICI‐BCG instead of BCG alone, one additional patient experienced a severe irAE (Figure 2).

## 4 DISCUSSION

In this systematic review and meta‐analysis of Phase III randomized controlled trials, we did not find a statistically significant improvement in high‐grade recurrence‐free survival among those receiving immune checkpoint inhibitor in addition to induction and maintenance BCG. On sensitivity analysis excluding the ALBAN trial, we found a 32% improvement in the risk of high‐grade recurrences. However, ICI‐based regimens were associated with almost a fivefold increase in Grade ≥3 TRAE. We did not find a clinically significant (<10‐point difference) decline in overall quality of life in the pooled analysis. On subgroup analysis, we did not find a clear subgroup that would benefit from this combination. Notably, the NNH with this ICI‐BCG combination was lower than NNT.

There is a need to decrease the recurrence/progression rate and the durability of response of therapies for patients with BCG‐naive HR NMIBC. Meta‐analysis increases the power of studies to detect an effect. Considering there were a small number of trials for which adjustment likely resulted in conservative estimates, the summary statistic may be misleading in this study. On sensitivity analysis excluding the ALBAN trial due to smaller sample size, definition of the primary endpoint, lower proportion of patients with T1 disease and patients with only‐CIS disease and non‐allowance of BCG reinduction for persistence of CIS at 3 months. The sensitivity analysis indicated that ICI‐based regimens were associated with a statistically significant 32% improvement in HG‐RFS. Although POTOMAC and CREST reported positive results, the results were likely driven by significant differences in recurrence of high‐grade NMIBC and not by progression to muscle invasive or distant disease [6, 7]. Further, in both trials, there was no difference noted in overall survival [6, 7]. However, longer follow‐up is required for the same considering the life expectancy for a patient with NMIBC may be >10 years [14]. In the pooled analysis, we found an approximately fivefold increase in odds of G3 + TRAE. A significant proportion of these are irAEs with NNH of 6–13 patients. In the CREST and POTOMAC trials, 20% and 45% of patients were prescribed corticosteroids [6, 7]. Considering significant toxicity, appropriate patient selection and counselling are important because the HG‐RFS improvement was likely driven by NMIBC recurrence and not progression requiring radical cystectomy for muscle invasive disease or other systemic therapy for metastatic disease. It is also important to consider the absolute effect sizes for both safety and efficacy outcomes during these discussions.

Considerations regarding the regimens themselves are also necessary to consider. Key differences between Sasanlimab and Durvalumab include the 2‐year versus 1‐year duration of ICI therapy, which may be associated with a higher risk of irAEs. Second, considering ICI is associated with a significant risk of irAEs and Durvalumab is given as an infusion (vs. subcutaneous administration of Sasanlimab), it is likely that a medical oncologist administers it who may be better trained to manage any AEs that arise.

On pooled analysis of the subgroup of patients with CIS, we found that patients without CIS were associated with a benefit with ICI‐based regimens. However, the CREST trial indicated enrichment among those with CIS disease. Because there is no biologically sound reason why CIS may respond to one ICI over the other both with similar mechanisms, this may arise due to the small size of subgroups, occurrence of pure or papillary‐concomitant CIS [15] or by variation in sampling for CIS based on gross cystoscopic appearance [16]. In the subgroup analysis of all trials and their pooled subgroup analysis, use of TICE strain of BCG was not associated with a significant improvement in HG‐RFS. We also found that female sex was not associated with an improvement in HG‐RFS. The lack of significance noted across several subgroups of these trials and in their pooled analysis may be attributed to the lack of the trials being powered for the subgroups. Considering the significant toxicity for relatively small improvement in HG‐RFS, a clear subgroup gaining benefit from combining ICI + BCG is not identifiable with the data available now. Considering these findings and lower NNH compared to NNT, patients should be appropriately counselled regarding the benefit reaped for the trade‐offs in toxicity.

In our pooled analysis, we did not find a clinically significant change in the overall QOL of patients. This is possible due to the small proportion of patients progressing to muscle invasive or metastatic disease for which more aggressive treatments are indicated. Other possible reasons could be due to the lack of specificity of the EORTC QLQ C30 questionnaire to capture issues that arise because of systemic immunotherapy despite an approximate fivefold increase in G3 + TRAE and significant steroid prescriptions (20% in CREST and 45% in POTOMAC) [17].

This study has limitations. First, the small number of trials limits the overall power, and H‐K adjustment may provide non‐statistically significant 95% CI [18]. However, POTOMAC and CREST reported statistically significant 95% CI for HG‐RFS. Second, there were differences in the BCG regimen and patient populations in the included trials. We accounted for this by sensitivity and subgroup analyses. Despite these limitations, this pooled analysis provides data for patient‐specific counselling on using ICI‐BCG (I + M) for patients with BCG‐naive HR‐NMIBC.

## 5 CONCLUSION

In this pooled analysis of randomized controlled trials, assessing the safety and efficacy of addition of immune checkpoint inhibitors to intravesical BCG for high‐risk non‐muscle invasive bladder cancer we did not find a statistically significant improvement in high‐grade recurrence free survival. However, on sensitivity analysis of two methodologically similar robust studies, we found a 32% improvement. Considering there was an approximately five‐fold increase in the risk of serious treatment‐related adverse events, further research is needed to clarify which patient subgroups may benefit the most from these regimens.

## AUTHOR CONTRIBUTIONS

**Renil S. Titus:** Conceptualization; methodology; software; formal analysis; data curation; writing—original draft; writing—review and editing; visualization. **Carlos Riveros:** Methodology; writing—original draft; writing—review and editing. **Eusebio Luna Velasquez:** Data curation; writing—original draft; writing—review and editing. **David‐Dan Nguyen:** Data curation; methodology; writing—review and editing. **Suran Somawardana:** Data curation; writing—review and editing. **Amar Singh:** Data curation; writing—review and editing. **Dharam Kaushik:** Methodology; writing—review and editing; supervision. **Guru Sonpavde:** Methodology; writing—review and editing; supervision. **Alexandre Zlotta:** Writing—review and editing; supervision. **Girish Kulkarni:** Writing—review and editing; supervision. **Srikala S. Sridhar:** Methodology; writing—original draft; writing—review and editing; supervision. **Christopher J. D. Wallis:** Methodology; writing—review and editing; supervision. **Raj Satkunasivam:** Writing—original draft; writing—review and editing; supervision; resources; project administration.

## CONFLICT OF INTEREST STATEMENT

Christopher J.D. Wallis has received consulting fees from Johnson & Johnson Innovative Medicine and Nanostics Inc.; honoraria/travel from AbbVie, Astellas, Astra Zeneca, Bayer, Johnson & Johnson Innovative Medicine, Knight Therapeutics, Intuitive Surgical, MashUP Media, Merck, Pfizer, Science & Medicine Canada, Sumitomo Pharmaceuticals, TerSera Canada, and Tolmar Pharmaceuticals Canada; and research funding from Astellas, Bayer, Knight Therapeutics, Sunnybrook HSC AFP, and Tolmar Pharmaceuticals. Srikala S. Sridhar has received consulting fees from AstraZeneca, Bayer, Bicycle Therapeutics, BMS, Daiichi Sankyo, EMD Serono, Gilead, Janssen, Merck, and Pfizer and research funding (institution) from Pfizer, Bayer, and EMD Serono. Raj Satkunasivam has received research funding or support to institution from Pfizer, Merck, UroGen, enGene, Photocure, Janssen, ProTARA, and CG Oncology and consulting fees from Pfizer (2022–2025), Intuitive Surgical (Proctor, 2019; 2023), and Ferring (2025).

## Supporting information


**Figure S1** Risk of bias among included studies per Cochrane RoB2.0 tool. LEGEND: D1–5: domains to assess risk of bias.
**Figure S2** Sensitivity analysis excluding ALBAN trial for HG‐RFS HG‐RFS: high grade recurrence free survival; SE: standard error; HR: hazard ratio; 95% CI: 95% confidence interval.
**Figure S3** Network plot for included studies in the network meta‐analysis. LEGEND: BCG(I + M): BCG induction and maintenance; IO: Immunotherapy.
**Figure S4** Subgroup analysis comparing ICI‐BCG(I ± M) v. BCG(I ± M). LEGEND: ICI‐BCG(I + M): immune checkpoint inhibitor combination with BCG induction and maintenance; HR: hazard ratio; 95% CI: 95% confidence interval; CIS: carcinoma in‐situ.
**Table S1:** Network meta‐analysis comparing HG‐RFS among different ICI‐BCG(I ± M) regimens. LEGEND: *Highest efficacy or toxicity or drop in QoL; TRAE: treatment related adverse events; EORTC QLQ C30: European Organisation for Research and Treatment of Cancer Quality of Life Questionnaire Core 30; BCG(I + M): BCG induction and maintenance.

## Data Availability

Data are not available, as this is a meta‐analysis that utilizes the published data of included studies.
