# Supplementary material for: Addition of immune checkpoint inhibitors to intravesical BCG for high‐risk BCG‐naïve non‐muscle invasive bladder cancer: Systematic review and meta‐analysis
Source: BJUI Compass. 2026 Mar 31;7(4):e70194. doi: 10.1002/bco2.70194 (PMC13098361; doi:10.1002/bco2.70194)
Supplement: Supplementary file 1 — Figure S1 Risk of bias among included studies per Cochrane RoB2.0 tool. LEGEND: D1–5: domains to assess risk of bias. Figure S2 Sensitivity analysis excluding ALBAN trial for HG‐RFS HG‐RFS: high grade recurrence free survival; SE: standard error; HR: hazard ratio; 95% CI: 95% confidence interval. Figure S3 Network plot for included studies in the network meta‐analysis. LEGEND: BCG(I + M): BCG induction and maintenance; IO: Immunotherapy. Figure S4 Subgroup analysis comparing ICI‐BCG(I ± M) v. BCG(I ± M). LEGEND: ICI‐BCG(I + M): immune checkpoint inhibitor combination with BCG induction and maintenance; HR: hazard ratio; 95% CI: 95% confidence interval; CIS: carcinoma in‐situ. Table S1: Network meta‐analysis comparing HG‐RFS among different ICI‐BCG(I ± M) regimens. LEGEND: *Highest efficacy or toxicity or drop in QoL; TRAE: treatment related adverse events; EORTC QLQ C30: European Organisation for Research and Treatment of Cancer Quality of Life Questionnaire Core 30; BCG(I + M): BCG induction and maintenance. [file BCO2-7-e70194-s001.docx]

New supplementary file

**Supplementary Figure-1 Risk of bias among included studies per Cochrane RoB2.0 tool**


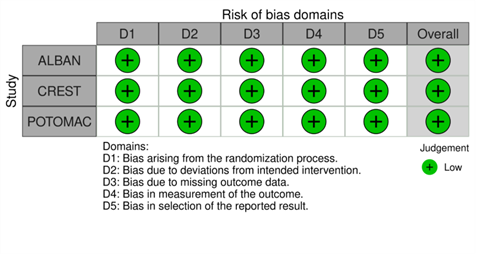


LEGEND: D1-5: domains to assess risk of bias

**Supplemental Figure-2 Sensitivity analysis excluding ALBAN trial for HG-RFS**
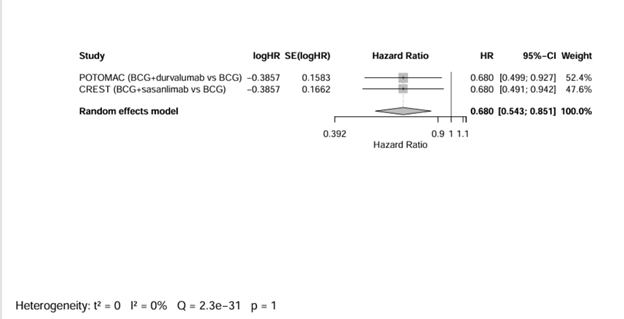
HG-RFS: high grade recurrence free survival; SE: standard error; HR: hazard ratio; 95%CI: 95% confidence interval

**Supplemental Figure-3 Network plot for included studies in the network meta-analysis**


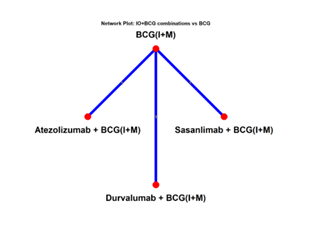


LEGEND: BCG(I+M): BCG induction and maintenance; IO: Immunotherapy

**Supplemental Figure-4 Subgroup analysis comparing ICI-BCG(I+M) v. BCG(I+M)**


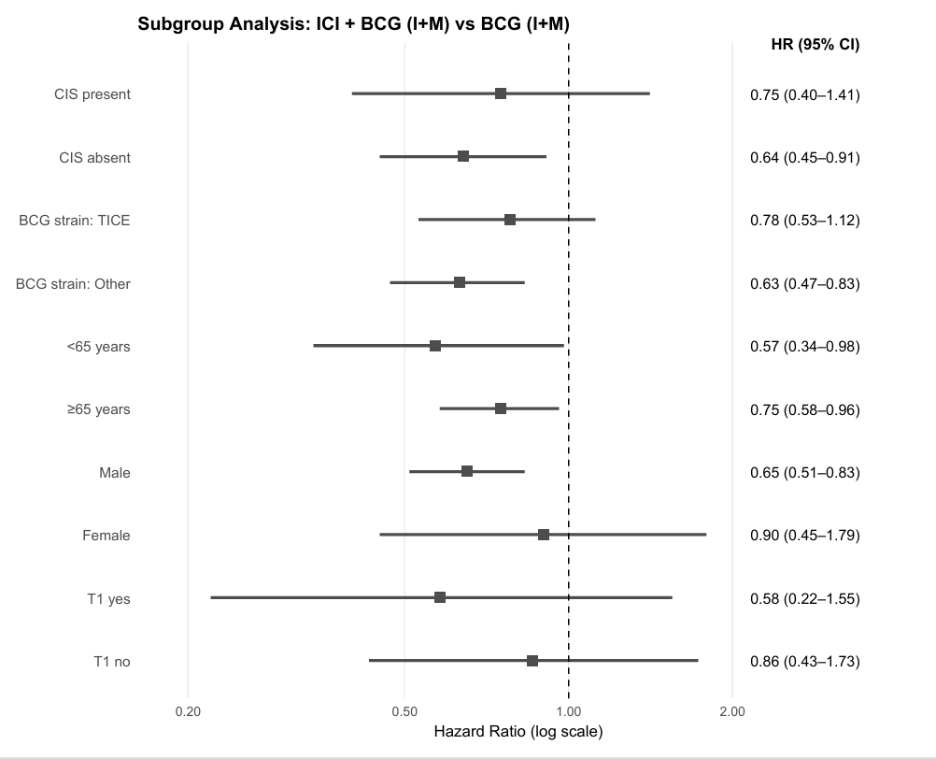


LEGEND: ICI-BCG(I+M): immune checkpoint inhibitor combination with BCG induction and maintenance; HR: hazard ratio; 95%CI: 95% confidence interval; CIS: carcinoma in-situ

**Supplementary Table-1: Network meta-analysis comparing HG-RFS among different ICI-BCG(I+M) regimens**

| Outcome | P-score | | | |
| --- | --- | --- | --- | --- |
|  | Durvalumab+BCG(I+M) | Sasanlimab+BCG(I+M) | Atezolizumab+BCG(I+M) | BCG(I+M) |
| Efficacy | **0.8184*** | 0.8165 | 0.2213 | 0.1528 |
| Grade ≥3 TRAE | **0.1779*** | 0.1786 | 0.6435 | 1.000 |
| Quality of Life (EORTC QLQ C30) | 0.5829 | **0.9109*** | NA | 0.0062 |

LEGEND: *Highest efficacy or toxicity or drop in QoL; TRAE: treatment related adverse events; EORTC QLQ C30: European Organisation for Research and Treatment of Cancer Quality of Life Questionnaire Core 30; BCG(I+M): BCG induction and maintenance
